# Application of Biomimetic Chromatography and QSRR Approach for Characterizing Organophosphate Pesticides

**DOI:** 10.3390/ijms26051855

**Published:** 2025-02-21

**Authors:** Katarzyna Ewa Greber, Karol Topka Kłończyński, Julia Nicman, Beata Judzińska, Kamila Jarzyńska, Yash Raj Singh, Wiesław Sawicki, Tomasz Puzyn, Karolina Jagiello, Krzesimir Ciura

**Affiliations:** 1 Department of Physical Chemistry, Faculty of Pharmacy, Medical University of Gdansk, Aleja Generała Józefa Hallera 107, 80-416 Gdansk, Poland; katarzyna.greber@gumed.edu.pl (K.E.G.); julianicman@gumed.edu.pl (J.N.); yash.singh@gumed.edu.pl (Y.R.S.); wieslaw.sawicki@gumed.edu.pl (W.S.); 2 Laboratory of Environmental Chemoinformatics, Faculty of Chemistry, University of Gdansk, Wita Stwosza 63, 80-308 Gdansk, Poland; k.topkaklonczynski.587@studms.ug.edu.pl (K.T.K.); beata.judzinska@phdstud.ug.edu.pl (B.J.); kamila.jarzynska@phdstud.ug.edu.pl (K.J.); tomasz.puzyn@ug.edu.pl (T.P.); 3 QSAR Lab, Trzy Lipy 3, 80-172 Gdańsk, Poland

**Keywords:** organophosphate, biomimetic chromatography, QSRR, artificial membrane chromatography

## Abstract

Biomimetic chromatography is a powerful tool used in the pharmaceutical industry to characterize the physicochemical properties of molecules during early drug discovery. Some studies have indicated that biomimetic chromatography may also be useful for the evaluation of toxicologically relevant molecules. In this study, we evaluated the usefulness of the biomimetic chromatography approach for determining the lipophilicity, affinity to phospholipids, and bind to plasma proteins of selected organophosphate pesticides. Quantitative structure–retention relationship (QSRR) models were proposed to understand the structural features that influence the experimentally determined properties. ACD/labs, Chemicalize, and alvaDesc software were used to calculate theoretical descriptors. Multilinear regression was used as the regression type, and feature selection was supported by a genetic algorithm. The obtained QSRR models were validated internally and externally, and they demonstrated satisfactory performance with key statistical parameters ranged from 0.844 to 0.914 for R^2^ and 0.696–0.898 for R^2^_ext_, respectively, indicating good predictive ability.

## 1. Introduction

The physicochemical properties of molecules are key factors influencing their adsorption, distribution, metabolism, elimination, and toxicity (ADMET) [1,2]. Therefore, determining their physicochemical properties plays a pivotal role in drug discovery and toxicological research, allowing the prediction of the behavior of molecules in the human body. Among the available methods, the chromatographic approach is superior because it allows automation of the process, eliminates the need for quantitative measurement, reduces sample size and organic solvent consumption, and allows measurement over a wider range. Consequently, chromatographic approaches have been widely applied in the pharmaceutical industry [3,4]. It has become the method of choice for screening tests based on the biochromatographic data prediction of complex pharmacokinetic properties of drugs, such as blood–brain barrier permeability [5], intestinal adsorption [6], the passage of drugs through the respiratory mucosa [7], the volume of distribution [8], and many others [9].

Recently, biomimetic chromatography has been applied to the characterization of toxicologically relevant substances, showing good agreement between chromatographic data and cellular and in vivo toxicity [10,11]. Similarly, some studies have indicated that biomimetic chromatography can be applied to predict the aquatic toxicity [4,12,13,14], bioconcentration [15], and ADME properties [16] of toxicologically relevant substances. However, the use of biomimetic chromatography in toxicological research is still in its infancy.

In this study, we demonstrated how integrating computational methods and biomimetic data enhances the understanding of the molecular characteristics of organophosphate pesticides, particularly regarding their interaction with crucial biomolecules such as phospholipids and plasma proteins (PP). To achieve this goal, the theoretical descriptors of quantitatively differentiated organophosphate pesticides were calculated. The target organophosphate pesticides were analyzed using three biomimetic chromatographic systems: C18-bonded reversed-phase, immobilized artificial membrane (IAM), and human serum albumin (HSA). A multiple linear regression (MLR) model was developed to establish the relationship between the molecular structure of organophosphate pesticides and retention indices related to their binding with biomolecules.

## 2. Results and Discussion

Organophosphates (OPs) are chemicals widely used in agrochemicals, including herbicides, pesticides, and insecticides. However, several aspects related to their safety are currently being considered and discussed. The most well-recognized mechanism of toxicity of OPs is the inhibition of acetylcholinesterase, which increases the neurotransmitter acetylcholine (ACh). This, in turn, affects nicotinic and muscarinic receptors and the central nervous system (CNS). The adverse effects of exposure to these substances include both acute and chronic toxicity, manifesting as adults neurotoxicity (ANT) and developmental neurotoxicity (DNT) [17,18,19].

Since the groundbreaking publication of Meyer [20], lipophilicity has been recognized as one of the most important factors determining the toxicity of chemical substances [21]. Lipophilicity, a compound’s affinity for lipids, is quantified using log*P*, which represents the logarithm of its partition coefficient between n-octanol and water. This system is favored for its resemblance to biological partitioning. While the traditional method for determining lipophilicity involves direct partitioning using the shake flask technique, it is both time-intensive and time-consuming. Therefore, indirect approaches, such as chromatography, are currently most commonly used to estimate lipophilicity. Chromatographic techniques offer further advantages, including the ability to utilize stationary phases coated with biomolecules, a method known as biochromatography or biomimetic chromatography.

Biochromatography originates in the pharmaceutical industry, with Valko and colleagues proposing this method to characterize potential drugs rapidly [22,23]. The affinity of the target molecules can be determined in a single rapid gradient experiment using stationary phases coated with biomolecules, like phospholipids and plasma proteins. Biochromatography offers several advantages, such as rapid analysis, small amounts of required substance, high reproducibility, and robustness, as the experiments are performed using HPLC. Biochromatography can also be utilized to analyze molecules with a very high affinity to PPs, which cannot be achieved using other analytical approaches, including spectroscopy (RLS, CD, UV-Vis, fluorimetry, IR, and NMR), and separation methods (equilibrium dialysis, ultrafiltration, binding with albumin microspheres) [24,25]. These advantages have made biomimetic chromatography popular in industry and academia [9,12,26,27].

In this study, we investigated a group of OPs to determine their affinity to biomolecules immobilized on chromatographic columns. The list of considered chemicals, along with their structures, is presented in Table S1. The biochromatographic data obtained are summarized in Table 1.

**Figure 1:**
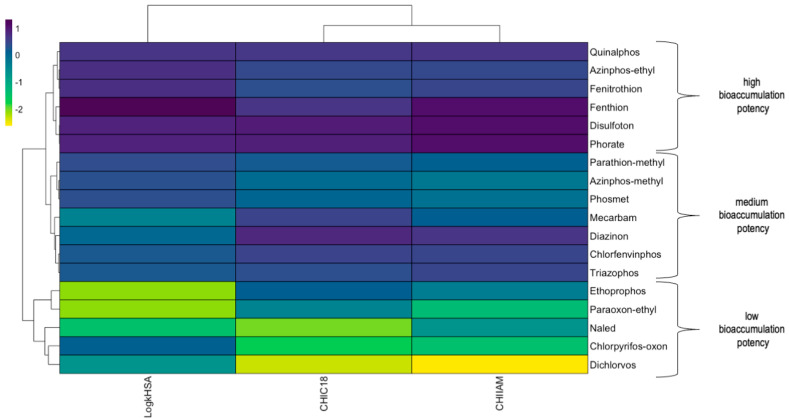
Heat map and hierarchical analysis based on biochromatographic data. The color indicates the affinity to biomolecules after auto-scaling, with dark purple indicating high binding and yellow indicating low binding.

Lipophilicity is a crucial factor that influences passive diffusion through biological membranes and determines their distribution and bioaccumulation. In this study, we used the CHI_C18_ scale from 0 to 100, as it allows easy interpretation of the results. The CHI_C18_ values varied considerably, with some compounds showing remarkably high lipophilicity, such as diazinon, disulfoton, and quinalphos, whereas others showed significantly lower values. Most molecules with low lipophilicity are low-molecular-weight structures without aromatic rings, such as dichlorvos or naled. Affinity to phospholipids, determined by IAM chromatography, showed moderate CHI_IAM_ values ranging from 16.56 to 42.43, which can be used as indicators of potential promiscuous binding and phospholipid interference. Among the compounds analyzed, phorate, fenthion, and disulfoton showed the highest CHI_IAM_ values, indicating their increased potential for membrane interactions. In addition, the PPB characteristics, expressed as %HSA, showed significant variation, with fenthion having the highest protein-binding capacity. High lipophilicity, phospholipids, and PPB affinity generally indicate a high risk of accumulation of target molecules in living organisms and are associated with higher toxicity and environmental impact. In the case of OPs, these properties are particularly important because OPs are neurotoxic, and lipophilicity is a known factor in blood–brain barrier transport. The chromatographic data were summarized and visualized as a heat map in Figure 1. Based on the result of the hierarchical analysis performed on the biochromoatgraphic measurement data matrix, three distinct groups of OPs are separated. These classes of OPs show different bioaccumulation potencies: low, medium, and high, respectively.

**Figure 2:**
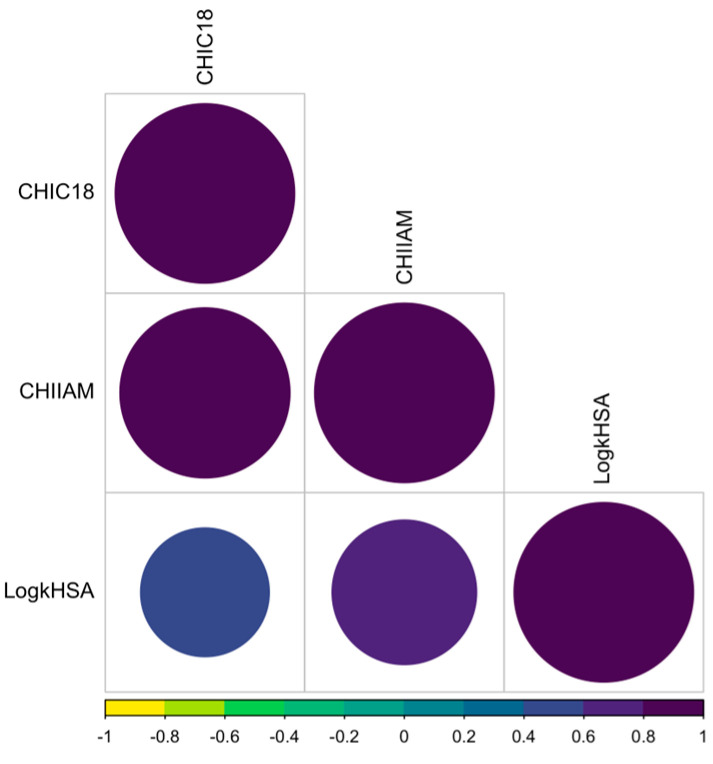
Correlation matrix between chromatographic lipophilicity, affinity to phospholipids, and HSA. The size and color refer to the correlation coefficient. A larger size and darker color indicates a higher correlation.

Because of the nature of chromatographically mimicked processes, high correlations are generally expected because lipophilicity is the main non-specific factor for binding to phospholipids and PP. However, in the case of HSA binding, we observed a relatively weak correlation (Figure 2). The most rational explanation is that some of the substances tested interact non-specifically with HSA and bind strongly to its pockets, which is consistent with thermodynamic, spectroscopic, and computational experiments [28,29,30].

To better understand the differences between lipophilicity and binding to phospholipids and HSA, a quantitative structure retention relationship (QSRR) approach was applied. QSRR was originally proposed by Kaliszan [31]. Starting from a simple prediction of the retention factor, this methodology was evaluated as a power computation tool applied in chromatographic science. One application of QSRR is to gain insight into the molecular mechanism in the chromatographic system under investigation, because QSRR models essentially create a bridge between the chemical structure of a compound and its properties, allowing us to understand which specific molecular features are responsible for its chromatographic behavior. To achieve this goal, we combined multiple linear regression (MLR) with a genetic algorithm (GA). GA is an optimization technique inspired by genetic principles and natural selection, using a heuristic approach to find the best solution for a problem. When integrating GA with regression methods, the process starts by generating an initial set of variables by randomly assigning binary values, with ‘1’ indicating inclusion and ‘0’ exclusion. The regression model is then applied to this variable set, and its effectiveness is evaluated. The model with superior performance is retained and serves as the basis for further genetic operations, such as crossover and mutation, according to the algorithm’s parameters. This approach has shown promise in previous studies for optimizing descriptor selection and improving model performance [32,33,34]. Table 2 summarizes the QSRR models obtained, together with the statistical figures. Table 3 lists the full names of each molecular descriptor used with its description and the block assigned to it.

**Figure 3:**
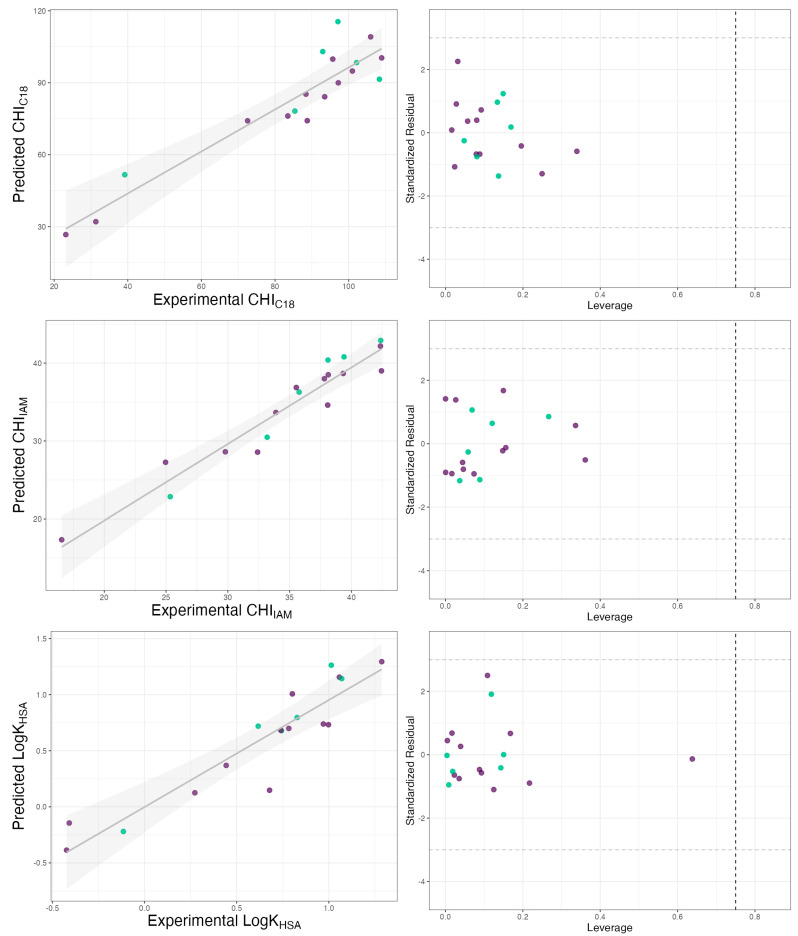
Comparison between experimental retention indices and those predicted by models and Williams plot for C18-bonded stationary phase, IAM stationary phase, and HAS column. Green dots refer to the training set and purple dots refer to the validation set.

The developed models showed acceptable statistical performance, including a good fit to the training data (R^2^, RMSE_tr_), internal validation (Q^2^_LOO_, RMSE_tr_), and external validation (R^2^_EXT_, RMSE_P_, CCC_Ext_), indicating their predictive power. In the case of CHI_C18_, external validation yielded the lowest R^2^_EXT_, and correspondingly, the RMSE_P_ error exhibited the highest value among the analyzed models. Nevertheless, all of the models obtained meet the criteria of Tropsha et al. (R^2^ > 0.6 and R^2^_EXT_, > 0.5) [35] and have similar statistical figures to other QSRR models published for datasets of similar size [33,36,37]. Williams plots (Figure 3) show that the molecules analyzed were in the model applicability domain (AD).

By analyzing the nature of the molecular descriptors present in each QSRR model, some conclusions can be drawn regarding the molecular mechanism of retention.

Model (1) highlights the role of electronic interactions and steric hindrance in lipophilicity. The positive contribution of SpMax_Dz(i), belonging to 2D matrix-based descriptors class, reflects the influence of the electronic properties, which enhance retention by increasing the ability of the compound to interact with the lipophilic phase. SpMax_Dz(i) is weighted by polarizability, which was previously combined with chromatographically measured lipophilicity [38]. In contrast, the negative contribution of R3s, a steric descriptor, indicated that bulky substituents reduced retention by limiting molecular accessibility to the stationary phase. The second model is related to the phospholipid binding. This model shows that both lipophilicity (LogP) and molecular branching (PJI3) significantly affect retention in systems that mimic phospholipid membranes. Higher LogP values increase hydrophobic interactions with the phospholipid phase, which aligns with the current state-of-the-art research [39]. The positive effect of PJI3, a descriptor quantifies a molecule’s geometric shape by assessing its deviation from an ideal spherical form, suggests that branched structures favor interactions with phospholipids. This model confirms that the interactions of OPs with phospholipids are more complex and go beyond classical lipophilicity and remain in line with our earlier observations underlining the importance of geometric descriptors [39].

The results of the third model represent the impact of the binding affinity of OPs to the HSA stationary phase, influenced by the intrinsic solubility (LogS) and topological distance-based descriptor (TDB05i). Conversely, negative LogS values indicate higher solubility, which contributes to reduced interactions with the binding sites. This observation has significant physical implications, as molecules with higher solubility demonstrate greater affinity for mobile phases, primarily composed of water. Furthermore, the influence of TDB05i reflects the impact of long-range electronic interactions on binding affinity.

## 3. Materials and Methods

### 3.1. Materials

#### 3.1.1. Analytical Standards

Analytical standards of OPs were obtained as follows: dichlorvos, triazophos, azinophos-ethyl, fenitrothion, mecarbam, ethion, fenthion, ethoprophos, parathion-methyl, dicrotophos, and disulfoton were obtained from Supelco (Steinheim, Germany); trichlorfon, phorat, and quinalphos were obtained from Honeywell (Charlotte, NC, USA); diazinon, chlorpyrifos, azinphos-methyl were obtained from VWR Chemicals (Radnor, PA, USA), phosmet was obtained from Cayman Chemical (Ann Arbor, MI, USA), and naled was obtained from Sigma-Aldrich (Steinheim, Germany). The 2D structures of all investigated OPs are presented in Figure S1.

Biochromatographic calibration was conducted utilizing the following reference substances: acetanilide, butyrophenone, diclofenac, and octanonophenone (Haverhill, MA, USA); acetophenone, benzimidazole, colchicine, indole, indometacin, paracetamol, and theophylline (Sigma-Aldrich, Steinheim, Germany); nicardipine and nizatidine (Cayman Chemical, Ann Arbor, MI, USA); carbamazepine, heptanophenone, hexanophenone, propiophenone, and valerophenone (Acros Organic, Pittsburg, PA, USA).

#### 3.1.2. Solvents

HPLC-grade solvents employed for the analyses were acetonitrile and isopropanol (Honeywell, Charlotte, NC, USA); deionized water (18.2 M'Ω), obtained using a Direct-Q 3 UV-R Ultrapure Water System (Millipore Corporation, Bedford, MA, USA); ammonium acetate buffer (VWR Chemicals, Radnor, PA, USA), and dimethyl sulfoxide (Avantor Performance Materials Poland S.A., Gliwice, Poland) used to dissolve samples.

### 3.2. Chromatographic Analysis

An HPLC system Prominence-1 LC-2030C 3D (Shimadzu, Tokyo, Japan) with a PDA detector was used. All biochromatographic experiments were conducted using the protocols proposed by Valko et al. [3,22,25,40], and were adopted in our laboratory [39,41,42].

The lipophilicity of the tested compounds was determined using a C18 XTERRA^®^ RP18 (3.5 μm, 4.6 × 150 mm) column (Waters, Milford, MA, USA). The affinity to human serum albumin was assessed using a CHIRALPACK^®^ HSA (5 μm, 4 × 50 mm) column (Daicel Chiral Technologies, West Chester, PA, USA). Affinity to phospholipids was examined using an IAM.PC.DD2 (4.6 × 100 mm) column (Regis Technologies, Morton Grove, IL, USA). Each column was equipped with an appropriate precolumn. Elution was conducted under the following conditions:

For the determination of lipophilicity, linear gradient 0–98% of phase B in 6.5 min, (phase A: 50 mM ammonium acetate, pH 7.4, phase B: acetonitrile), maintaining a mobile phase flow rate of 1.5 mL/min and column oven temperature of 30 °C.

For the determination of human serum albumin affinity, a linear gradient of 0–20% of phase B for 15 min, 20% of phase B for 12 min, and 20–0% of phase B for 5 min (phase A: 50 mM ammonium acetate, pH 7.4, phase B: isopropanol) was used, maintaining a mobile phase flow rate of 0.9 mL/min and column oven temperature of 30 °C.

For the determination of phospholipid affinity, linear gradient 0–85% of phase B in 5.25 min, 85% of phase B for 0.5 min (phase A: 50 mM ammonium acetate, pH 7.4, phase B: acetonitrile), maintaining a mobile phase flow rate of 1.5 mL/min, and column oven temperature of 30 °C.

The sample preparation involved dissolving the substance of interest in dimethyl sulfoxide to obtain a solution with a concentration of 100 µg/mL. The injected volume was 10 μL. The detection was performed at a wavelength of 254 nm. Each sample was analyzed three times. The raw retention data are listed in Table S1–S3.

### 3.3. Molecular Descriptors

Molecular descriptors were computed utilizing Chemicalize software (https://chemicalize.com, accessed on 1 February 2024) and alvaDesc software (version 2.0.10, Alvascience, Lecco, Italy). They were based on the geometry optimization achieved by Baker’s Eigen implemented in MOPAC software (version 3.0). Subsequently, descriptors characterized as constant, nearly constant, or exhibiting high correlation (r = 0.95) were eliminated from the dataset. Subsequently, descriptors characterized as constant, nearly constant, or exhibiting high correlation (r = 0.95) were eliminated from the dataset. The total number of descriptors was 3170. Moreover, various lipophilicity indices for each compound were derived using the SwissADME web application (http://www.swissadme.ch, accessed on 1 February 2024) based on the respective SMILES representations.

### 3.4. QSRR Model Development and Validation

The selection of descriptors was optimized by applying a genetic algorithm (GA). Subsequently, a multiple linear regression (MLR) analysis was conducted using a self-programmed Python script. The parameters for the GA included a population size of 10, mutation rate of 20, and a total of 500 generations. Before implementing GA-MLR for each modeled endpoint, the target solutes were systematically divided into a training set (*n* = 12) and a validation set (*n* = 6). The LogK**_HSA_** endpoint was specifically analyzed using two previously assessed endpoints, CHI**_C18_** and CHI**_IAM_**, as descriptors.

We validated our QSRR models according to the Organization for Economic Cooperation and Development (OECD) standards using various metrics to evaluate their performance, including goodness of fit (coefficient of determination *R*^2^, root mean square calibration error *RMSE_TR_*), robustness (leave-one-out cross-validation *Q*^2^*_LOO_*, root mean square error of leave-one-out cross-validation *RMSE_LOO_*), and predictive ability (external validation coefficient *R*^2^*_EXT_*, root mean square error of prediction RMSE_P_, concordance correlation coefficient *CCC_EXT_*), calculated as the following [43,44]:

(1) $$R^{2}=1-\frac{\sum {\left(y_{pred}-y_{obs}\right)}^{2}}{\sum {\left(y_{obs}-{\bar{y}}_{obs}\right)}^{2}}$$

(2) $${RMSE}_{TR}=\sqrt{\frac{\sum {\left(y_{pred}-y_{obs}\right)}^{2}}{n_{TR}}}$$

(3) $$Q_{LOO}^{2}=1-\frac{\sum {\left(y_{cv}-y_{obs}\right)}^{2}}{\sum {\left(y_{obs}-{\bar{y}}_{obs}\right)}^{2}}$$

(4) $${RMSE}_{LOO}=\sqrt{\frac{\sum {\left(y_{cv}-y_{obs}\right)}^{2}}{n_{TR}}}$$

(5) $$Q_{EXT}^{2}=1-\frac{\sum {\left(y_{pred}-y_{obs}\right)}^{2}}{\sum {\left(y_{obs}-{\bar{y}}_{obs}\right)}^{2}}$$

(6) $${RMSE}_{EXT}=\sqrt{\frac{\sum {\left(y_{pred}-y_{obs}\right)}^{2}}{n_{EXT}}}$$

(7) $${CCC}_{EXT}\;=1-\frac{2\sum \left(y_{obs}-{\bar{y}}_{obs}\right)(y_{pred}-{\bar{y}}_{pred})}{\sum {\left(y_{obs}-{\bar{y}}_{obs}\right)}^{2}+\sum {\left(y_{pred}-{\bar{y}}_{pred}\right)}^{2}+n_{EXT}{\left(y_{pred}-{\bar{y}}_{pred}\right)}^{2}}$$

where $y_{obs},\;y_{cv}$ and $y_{pred}$ are experimental, estimated in the cross-validation process, and externally predicted values of the modeled variable, respectively; ${\bar{y}}_{obs}$, ${\bar{y}}_{cv}$, and ${\bar{y}}_{pred}$ are the average values of the modeled variable, respectively; and $n_{TR}$ and $n_{EXT}$ are training and prediction sets [44].

A good QSRR model should exhibit high values for the *R*^2^, *Q*^2^*_LOO_*, and *R*^2^*_EXT_* metrics while ensuring low and comparable error rates. Error-based metrics are essential, as significant discrepancies may indicate model overfitting, which could restrict its generalizability [44].

Reliable predictions of the models are delineated by boundaries known as the applicability domain (AD). These boundaries were established by plotting the leverage coefficients (*h*) and standardized residuals (*y_pred_* − *y_obs_*) on the Williams plot. The leverage coefficients indicate the structural similarity of the compounds to the training set. The critical leverage value was calculated using the following formula:

(8) $$h^{*}=3pn^{-1}$$

where *p* represents the number of descriptors and *n* denotes the number of compounds in the training set. Compounds that exceeded this critical value were deemed to be outside the applicability domain. Additionally, standardized residuals are expected to remain within three standard deviations, thereby reflecting the model’s predictive accuracy [44].

## 4. Conclusions

This investigation demonstrated the efficacy of biomimetic chromatography and QSRR modeling for the characterization of OPs. Biomimetic chromatography yielded significant data regarding lipophilicity, phospholipid affinity, and protein binding of OP pesticides in a short time, and as an indirect method, it did not require large quantities of reagents. Biochromatographic data revealed substantial variations in the physicochemical properties of OPs, facilitating classification into low, medium, and high bioaccumulation potential categories. Furthermore, the integration of computational methods with biomimetic data enhances our understanding of organophosphate interactions with biomolecules. The developed QSRR models established strong correlations between molecular structure and chromatographic retention, elucidating retention mechanisms. Nevertheless, given the restricted sample size of the examined group, the findings should be approached with caution. This study underscores the complexity of OPs interactions with biological systems, beyond classical lipophilicity considerations. Furthermore, it highlights the need for additional investigation and dialog among researchers and regulatory authorities to enhance the relevance of biomimetic chromatography as a tool for regulatory purposes. For the assessment of PPB, we do not have a standardized procedure that is universally accepted. The variability of columns coated with proteins or phospholipids can be effectively controlled through the utilization of calibration mixtures, which mitigate the influence of retention time deviations resulting from factors such as the natural aging of these columns. However, in the case of columns with immobilized C_18_ groups, the diversity of types and significant differences in silica functionalization, particle size, and other parameters necessitate additional adjustments.

## Figures and Tables

**Table 1:** Summary of the chromatographic indices of the target compounds.

No.	Name	CHI_C18_	CHI_IAM_	Log*k*_HSA_	%HSA
1	Azinphos-ethyl	95.69	37.81	1.01	92.07
2	Azinphos-methyl	83.47	33.18	0.78	86.70
3	Chlorfenvinphos	97.18	38.13	0.74	85.54
4	Chlorpyrifos-oxon	39.17	24.96	0.68	83.49
5	Diazinon	105.98	39.34	0.62	81.33
6	Dichlorvos	23.17	16.56	0.27	65.86
7	Disulfoton	108.97	42.34	1.06	92.86
8	Ethoprophos	88.39	32.41	−0.41	28.35
9	Fenitrothion	93.49	38.10	1.00	91.78
10	Fenthion	102.16	42.37	1.29	96.04
11	Mecarbam	97.09	35.77	0.44	74.23
12	Naled	31.32	29.79	−0.11	43.86
13	Paraoxon-ethyl	72.54	25.34	−0.42	27.68
14	Parathion-methyl	88.75	35.54	0.83	87.94
15	Phorate	108.36	42.43	1.07	93.08
16	Phosmet	85.39	33.89	0.80	87.23
17	Quinalphos	101.01	39.40	0.97	91.23
18	Triazophos	92.96	38.08	0.74	85.43

CHI_C18_—chromatographic hydrophobicity index of RP-HPLC; CHI_IAM_—chromatographic hydrophobicity index of IAM-HPLC; Log*k*_HSA_—chromatographic determines affinity do HAS-HPLC; %HSA—of binding to HAS.

**Table 2:** Obtained QSRR models with statistical figures.

Model	Equation							
1	CHI_C18_ = 82.496 + 17.488 SpMax_Dz(i) − 17.080 R3s							
2	CHI_IAM_ = 34.273 + 4.836 LogP + 3.392 PJI3							
3	Log*k*_HSA_ = 0.600 − 0.423 LogS − 0.284 TDB05i							
	R^2^	RMSE_TR_	Q^2^_LOO_	RMSE_LOO_	R^2^_EXT_	RMSE_P_	CCC_EXT_	
1	0.870	6.889	0.894	8.622	0.696	12.552	0.833	
2	0.914	2.008	0.855	2.735	0.880	1.884	0.955	
3	0.844	0.217	0.576	0.340	0.898	0.125	0.960	

**Table 3:** Full names and blocks of molecular descriptors applied in QSRR analysis.

Model	Name	Description	Block	Software
1	SpMax_Dz(i)	Leading eigenvalue from Barysz matrix weighted by ionization potential	2D matrix-based descriptors	AlvaDesk
	R3s	R autocorrelation of lag 3/weighted by I-state	GETAWAY descriptors	AlvaDesk
2	LogP	Calculates the logarithm of the octanol/water partition coefficient	Physicochemical properties	Chemicalize
	PJI3	3D Petitjean shape index	Geometrical descriptors	AlvaDesk
3	LogS	Intrinsic solubility	Physicochemical properties	Chemicalize
	TDB05i	Topological distance-based descriptors—lag 5 weighted by ionization potential	3D autocorrelations	AlvaDesk

## Data Availability

Data available as Supplementary Materials.

## Supplementary Materials

The following supporting information can be downloaded at: https://www.mdpi.com/article/10.3390/ijms26051855/s1.
