# Supplementary material for: Application of Biomimetic Chromatography and QSRR Approach for Characterizing Organophosphate Pesticides
Source: Int J Mol Sci. 2025 Feb 21;26(5):1855. doi: 10.3390/ijms26051855 (PMC11898958; doi:10.3390/ijms26051855)
Supplement: Supplementary file 1 [file ijms-26-01855-s001.zip › ijms-3441047-supplementary.pdf]

*Supplementary Materials*

# Application of Biomimetic Chromatography and QSRR Approach for Characterizing Organophosphate Pesticides

**Katarzyna Ewa Greber [1,†], Karol Topka Kłończyński [2,†], Julia Nicman [1], Beata Judzińska [2], Kamila Jarzyńska [2], Yash Raj Singh [1], Wiesław Sawicki [1], Tomasz Puzyn [2,3], Karolina Jagiello [2,3,*] and Krzesimir Ciura [2,3,*]**

1 Department of Physical Chemistry, Faculty of Pharmacy, Medical University of Gdansk, Aleja Generała Józefa Hallera 107, 80-416 Gdansk, Poland; katarzyna.greber@gumed.edu.pl (K.E.G.); julianicman@gumed.edu.pl (J.N.); yash.singh@gumed.edu.pl (Y.R.S.); wieslaw.sawicki@gumed.edu.pl (W.S.)

2 Laboratory of Environmental Chemoinformatics, Faculty of Chemistry, University of Gdansk, Wita Stwosza 63, 80-308 Gdansk, Poland; k.topkaklonczynski.587@studms.ug.edu.pl (K.T.K.); beata.judzinska@phdstud.ug.edu.pl (B.J.); kamila.jarzynska@phdstud.ug.edu.pl (K.J.); tomasz.puzyn@ug.edu.pl (T.P.)

3 QSAR Lab, Trzy Lipy 3, 80-172 Gdańsk, Poland

* Correspondence: karolina.jagiello@ug.edu.pl (K.J.); krzesimir.ciura@ug.edu.pl (K.C.)

† These authors contributed equally to this work.

Figure S1. 2D strucutures of investigated OPs

| No. | Structural | Name |
|---|---|---|
| 1 | 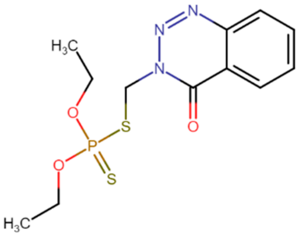 | Azinphos-ethyl |
| 2 | 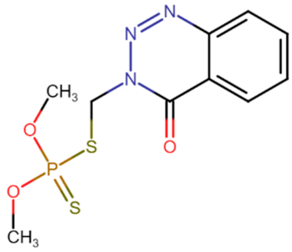 | Azinphos-methyl |
| 3 | 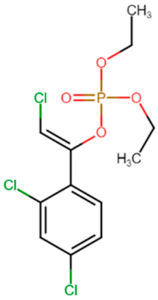 | Chlorfenvinphos |
| 4 | 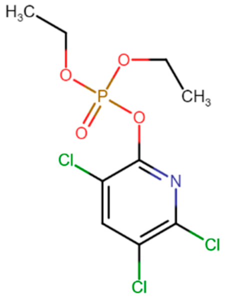 | Chlorpyrifos-oxon |
| 5 | 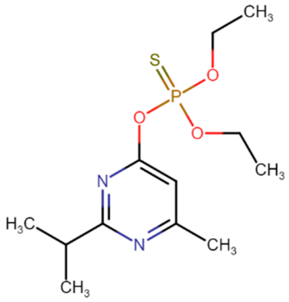 | Diazinon |

| | | |
|---|---|---|
| 6 | 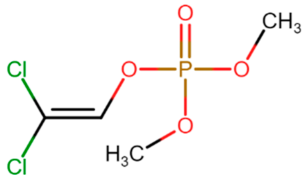 | Dichlorvos |
| 7 | 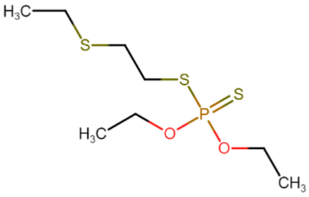 | Disulfoton |
| 8 | 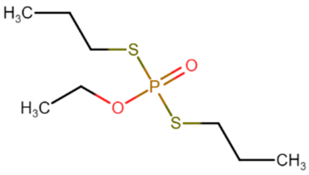 | Ethoprophos |
| 9 | 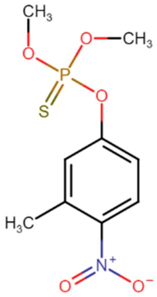 | Fenitrothion |
| 10 | 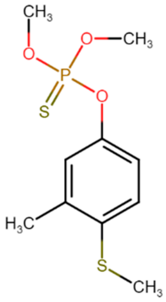 | Fenthion |
| 11 | 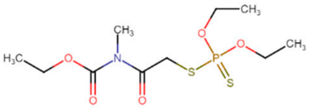 | Mecarbam |

| | | |
|---|---|---|
| 12 | 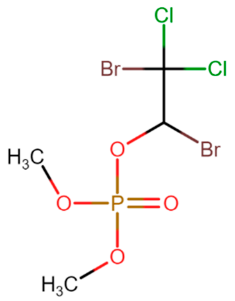 | Naled |
| 13 | 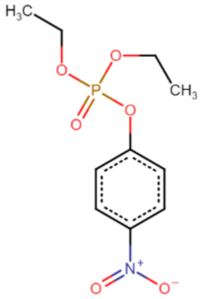 | Paraoxon-ethyl |
| 14 | 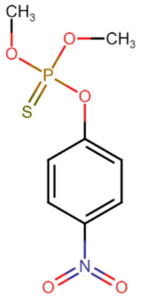 | Parathion-methyl |
| 15 | 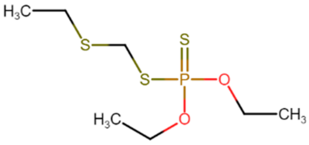 | Phorate |
| 16 | 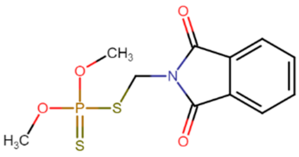 | Phosmet |
| 17 | 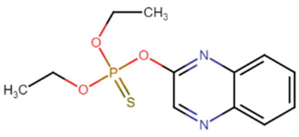 | Quinalphos |

| | | |
|---|---|---|
| 18 | 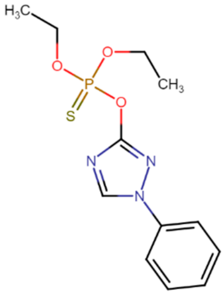 | Triazophos |

Table S1. Retention time of investigated OPs for HSA column

| **Name** | **$t_1$** | **$t_2$** | **$t_3$** | **$t_{mean}$** | **SD** | **logT** |
|---|---|---|---|---|---|---|
| Azinphos-ethyl | 12,72 | 12,89 | 12,82 | 12,81 | 0,08 | 1,11 |
| Azinphos-methyl | 10,07 | 9,75 | 9,95 | 9,92 | 0,16 | 1,00 |
| Chlorfenvinphos | 9,44 | 9,58 | 9,48 | 9,50 | 0,07 | 0,98 |
| Chlorpyrifos-oxon | 8,82 | 8,87 | 8,84 | 8,84 | 0,03 | 0,95 |
| Diazinon | 8,20 | 8,31 | 8,27 | 8,26 | 0,05 | 0,92 |
| Dichlorvos | 5,74 | 5,59 | 5,62 | 5,65 | 0,08 | 0,75 |
| Disulfoton | 13,24 | 13,65 | 13,43 | 13,44 | 0,20 | 1,13 |
| Ethoprophos | 2,66 | 2,65 | 2,66 | 2,66 | 0,01 | 0,42 |
| Fenitrothion | 12,44 | 12,77 | 12,57 | 12,59 | 0,16 | 1,10 |
| Fenthion | 17,18 | 17,46 | 17,35 | 17,33 | 0,14 | 1,24 |
| Mecarbam | 6,78 | 6,85 | 6,81 | 6,82 | 0,03 | 0,83 |
| Naled | 3,65 | 3,73 | 3,65 | 3,68 | 0,05 | 0,57 |
| Paraoxon-ethyl | 2,60 | 2,64 | 2,60 | 2,62 | 0,02 | 0,42 |
| Parathion-methyl | 10,37 | 10,52 | 10,41 | 10,43 | 0,07 | 1,02 |
| Phorate | 13,36 | 13,85 | 13,70 | 13,63 | 0,25 | 1,13 |
| Phosmet | 10,20 | 10,11 | 10,10 | 10,14 | 0,06 | 1,01 |
| Quinalphos | 12,11 | 12,24 | 12,29 | 12,21 | 0,09 | 1,09 |
| Triazophos | 9,54 | 9,36 | 9,48 | 9,46 | 0,09 | 0,98 |

Table S2. Retention time of investigated OPs for IAM column

| **Name** | **$t_1$** | **$t_2$** | **$t_3$** | **$t_{mean}$** | **SD** |
|---|---|---|---|---|---|
| Azinphos-ethyl | 4,24 | 4,25 | 4,25 | 4,25 | 0,01 |
| Azinphos-methyl | 3,95 | 3,96 | 3,95 | 3,95 | 0,00 |
| Chlorfenvinphos | 4,27 | 4,26 | 4,27 | 4,27 | 0,00 |
| Chlorpyrifos-oxon | 3,40 | 3,47 | 3,43 | 3,43 | 0,03 |
| Diazinon | 4,29 | 4,39 | 4,35 | 4,34 | 0,05 |
| Dichlorvos | 2,91 | 2,90 | 2,91 | 2,90 | 0,00 |
| Disulfoton | 4,53 | 4,54 | 4,53 | 4,53 | 0,00 |
| Ethoprophos | 3,90 | 3,91 | 3,90 | 3,90 | 0,00 |
| Fenitrothion | 4,24 | 4,29 | 4,28 | 4,27 | 0,03 |

| | | | | | |
|---|---|---|---|---|---|
| Fenthion | 4,53 | 4,54 | 4,53 | 4,53 | 0,01 |
| Mecarbam | 4,10 | 4,14 | 4,12 | 4,12 | 0,02 |
| Naled | 3,69 | 3,79 | 3,75 | 3,74 | 0,05 |
| Paraoxon-ethyl | 3,44 | 3,47 | 3,47 | 3,46 | 0,02 |
| Parathion-methyl | 4,10 | 4,10 | 4,10 | 4,10 | 0,00 |
| Phorate | 4,53 | 4,54 | 4,54 | 4,54 | 0,00 |
| Phosmet | 4,00 | 4,00 | 4,00 | 4,00 | 0,00 |
| Quinalphos | 4,35 | 4,35 | 4,35 | 4,35 | 0,00 |
| Triazophos | 4,28 | 4,24 | 4,26 | 4,26 | 0,02 |

Table S3. Retention time of investigated OPs for C18 column

| **Name** | $t_1$ | $t_2$ | $t_3$ | $t_{mean}$ | **SD** |
|---|---|---|---|---|---|
| Azinphos-ethyl | 5,94 | 5,94 | 5,92 | 5,93 | 0,01 |
| Azinphos-methyl | 5,44 | 5,44 | 5,41 | 5,43 | 0,02 |
| Chlorfenvinphos | 6,00 | 6,00 | 5,99 | 6,00 | 0,01 |
| Chlorpyrifos-oxon | 3,60 | 3,60 | 3,61 | 3,60 | 0,01 |
| Diazinon | 6,38 | 6,37 | 6,35 | 6,36 | 0,01 |
| Dichlorvos | 2,93 | 2,93 | 2,91 | 2,92 | 0,01 |
| Disulfoton | 6,49 | 6,49 | 6,45 | 6,48 | 0,02 |
| Ethoprophos | 5,64 | 5,64 | 5,65 | 5,64 | 0,01 |
| Fenitrothion | 5,85 | 5,85 | 5,81 | 5,84 | 0,02 |
| Fenthion | 6,21 | 6,21 | 6,19 | 6,20 | 0,01 |
| Mecarbam | 6,00 | 6,00 | 6,01 | 6,00 | 0,01 |
| Naled | 3,27 | 3,27 | 3,28 | 3,27 | 0,01 |
| Paraoxon-ethyl | 5,00 | 4,98 | 4,97 | 4,98 | 0,01 |
| Parathion-methyl | 5,66 | 5,65 | 5,64 | 5,65 | 0,01 |
| Phorate | 6,47 | 6,47 | 6,45 | 6,46 | 0,01 |
| Phosmet | 5,51 | 5,51 | 5,49 | 5,51 | 0,01 |
| Quinalphos | 6,16 | 6,16 | 6,12 | 6,15 | 0,02 |
| Triazophos | 5,83 | 5,83 | 5,81 | 5,82 | 0,01 |
